# Is there an association between intimate partner violence and the prevalence of cervical cancer screening in Jordan?

**DOI:** 10.1371/journal.pone.0290678

**Published:** 2023-08-31

**Authors:** Grace Urquhart, Sara J. Maclennan, Aravinda Meera Guntupalli

**Affiliations:** 1 Queen Elizabeth Health NHS Foundation Trust, Gateshead, Newcastle, United Kingdom; 2 University of Aberdeen, Aberdeen, United Kingdom; 3 Institute of Applied Health Sciences, University of Aberdeen, Aberdeen, United Kingdom; University of Ghana Medical School, GHANA

## Abstract

**Background:**

Major health inequalities exist surrounding the utilisation of cervical cancer screening services globally. Jordan, a low- and middle-income country, has poor screening rates (15.8%), with barriers to accessing services, including lack of education. Emerging studies demonstrate that intimate partner violence (IPV) impacts reproductive health decisions. As a large proportion of Jordanian women have reported experiencing IPV, this study examines the association between IPV and cervical cancer screening in Jordan, the first of its kind using national-level data.

**Methods:**

Using Jordan’s Demographic Health Survey 2017–18, cervical cancer screening awareness and self-reported screening were estimated in participants who answered questions on IPV (n = 6679). After applying sample weights, Heckman’s two-stage probit model determined the association of awareness and utilisation of cervical cancer screening with experience of IPV, adjusting for the socio-economic factors.

**Results:**

Of the women with privacy to answer the IPV module, 180 (3.4%) were found to be victims of sexual violence, 691 of physical violence (12.6%) and 935 (16.2%) of emotional violence. Women subjected to sexual violence were less likely to admit to having awareness of a Pap smear test; however, this did not impact screening rates. Victims of emotional violence were more likely to be screened than non-victims. No association between physical violence and cervical cancer screening was found.

**Conclusions:**

A significant association between cervical screening awareness and IPV demonstrates that cancer screening policies must consider IPV among women to improve screening awareness. The paper further sheds light on the paradoxical association between emotional violence and screening. It is acknowledged this situation may be far worse than reported, as women without autonomy were unlikely to answer IPV questions that may endanger them—targeted surveys on cervical cancer screening warrant further investigation.

## Introduction

Cervical cancer is the fourth most common woman’s cancer worldwide and one of the most successfully treatable if detected early. Most cervical cancer cases are caused by the sexually transmitted infection Human Papilloma Virus (HPV) type 16 and/or 18. Currently, major health inequalities surround the utilisation of cervical cancer screening services globally [1]. It is widely recognised that low- and middle-income countries (LMICs) have poor cervical cancer screening rate [2]. Recent estimates suggest that 84% of women aged 30–49 years living in high-income countries have been screened for cervical cancer ever in their lifetime, compared with 48% in upper middle, 9% lower-middle income countries [3].

In recent years The World Health Organisation (WHO) have adapted screening recommendations to include HPV DNA testing in woman aged 30, with regular screening every 5 to 10 years [4]. However, a recent study showed that only 48 countries, the majority of whom are high or upper-middle income countries, have adopted or are planning to adopt HPV-based screening [3]. The primary cervical cancer screening test for LMICs is the opportunistic ‘Pap smear test’ involving visual cervix inspection to identify both precancerous cell changes caused by HPV and early-stage cancer development [4, 5]. Despite relying on opportunistic methods that are often unreliable, LMICs have poorer coverage due to weaker health infrastructure and lower screening participation. To make matters worse, only 49% of low and lower-middle income countries have official recommendations to screen for cervical malignancy. Without these early detection methods and recommendations, the World Health Organisation’s (WHO) cervical cancer elimination strategy for 2030–70% of girls screened by age 35—will be impossible [6].

Poor screening is also apparent in the low- and middle-income country Jordan, where females comprise 49.5% of the predominantly young 10.2 million population. Of the female population, 62% are aged between 15–65 years old [7, 8]. Jordan’s crude incidence rate of cervical cancer is 2.3 per 100,000 women [9] with an estimated 277 new cases in 2020 [10], however, this incidence rate may be unreliable due to incomplete registration. Furthermore, the lack of awareness and access amongst Jordanian women to the costly HPV vaccine, an intervention to avert the development of cervical cancer [11, 12] may be associated with higher death rates.

Unlike several high-income countries, Jordan has no structured national screening programme and instead, screening is provided in public practice to those aged 25–35 free of charge by patient demand or opportunistically at appointments. This service does not actively issue invitations and lacks quality assurance allowing monitoring and evaluation of impact [13]. Conflicting Jordanian sourced evidence suggests screening from the age of 21, or from the initiation of sexual intercourse [14, 15], however, a cancer centre in the Capital city Amman recommends screening from three years post first sexual intercourse [16]. The lack of recommendations and incoherent practices result in low cervical cancer screening rates. Other barriers to accessing screening in Jordan include a lack of encouragement from healthcare providers, a preference for female healthcare staff and limited health education and promotion [7]. Furthermore, perceived barriers may include embarrassment, fear, or pain [17]. One emerging factor to consider is the association with intimate partner violence (IPV), defined by the WHO as ’any behaviour that causes physical, psychological or sexual harm to those in the relationship’ [18]. IPV is a global health concern, notably in Jordan, with a high prevalence and acceptance within a traditionally patriarchal society where males and women living with extended family or rurally are more accepting towards this behaviour [19].

Aggravation of stress and depressive symptoms brought on by this form of violence may impact lifestyle changes such as smoking [20] which is an identified risk factor for the development of cervical cancer cells. IPV is also linked to high-risk sexual behaviours, including non-condom usage and exposure to sexually transmitted diseases which can also play a role in the aetiology of cervical cancer [21–23]. Furthermore, studies show that victims of IPV are diagnosed with cervical cancer at a younger age than the general population of women who have not been subject to IPV [24].

Alongside IPV having been linked to adverse health outcomes including cervical cancer, it may also be associated with poor use of health services including cancer screening. Woman may be faced with the physical barrier of being unable to access care by their partners who control many aspects of their life and wellbeing. IPV related barriers to accessing care also include fear of flashbacks, pain, mistrust, or embarrassment associated with male healthcare providers [25, 26].

Most IPV research focuses on the health impact of physical and/or sexual violence, although recent research increasingly focuses on emotional or psychological abuse [27, 28]. In Jordan, IPV has been shown to interfere with decisions surrounding modern contraceptive use and termination of pregnancy [29]. But no study has looked at the association of IPV with cervical cancer screening. Global literature suggests that IPV is associated with severe short and long-term psychological and physical health consequences, significantly impacting a woman’s health outcomes and healthcare access. Hence his project aims to address gaps in research to better understand if IPV is identified as a predictor of cervical cancer screening in Jordan. This is the first study of its kind to use this nationwide data in this context, with the ultimate aim to provide recent data that can be used in future to assist policymakers when addressing historically low cervical cancer screening rates.

## Material and methods

### Data source and sampling

This secondary data analysis used Jordan’s nationally representative and anonymised cross-sectional 2017–2018 Demographic Health Survey (DHS) which was accessed from the official DHS website [14]. A two-stage stratified sample was selected from the 2015 census, each governorate separated into urban and rural areas, with 26 sampling strata constructed in total. 970 clusters were selected at the first stage with households listed in all selected clusters. During stage two, 20 households per cluster were selected: all ever-married women aged 15–49 identified as residents of the selected households or visitors of these the night before the survey were eligible. This was translated into Arabic from the original English questionnaire design, with only one eligible woman per household selected.

**Fig 1:**
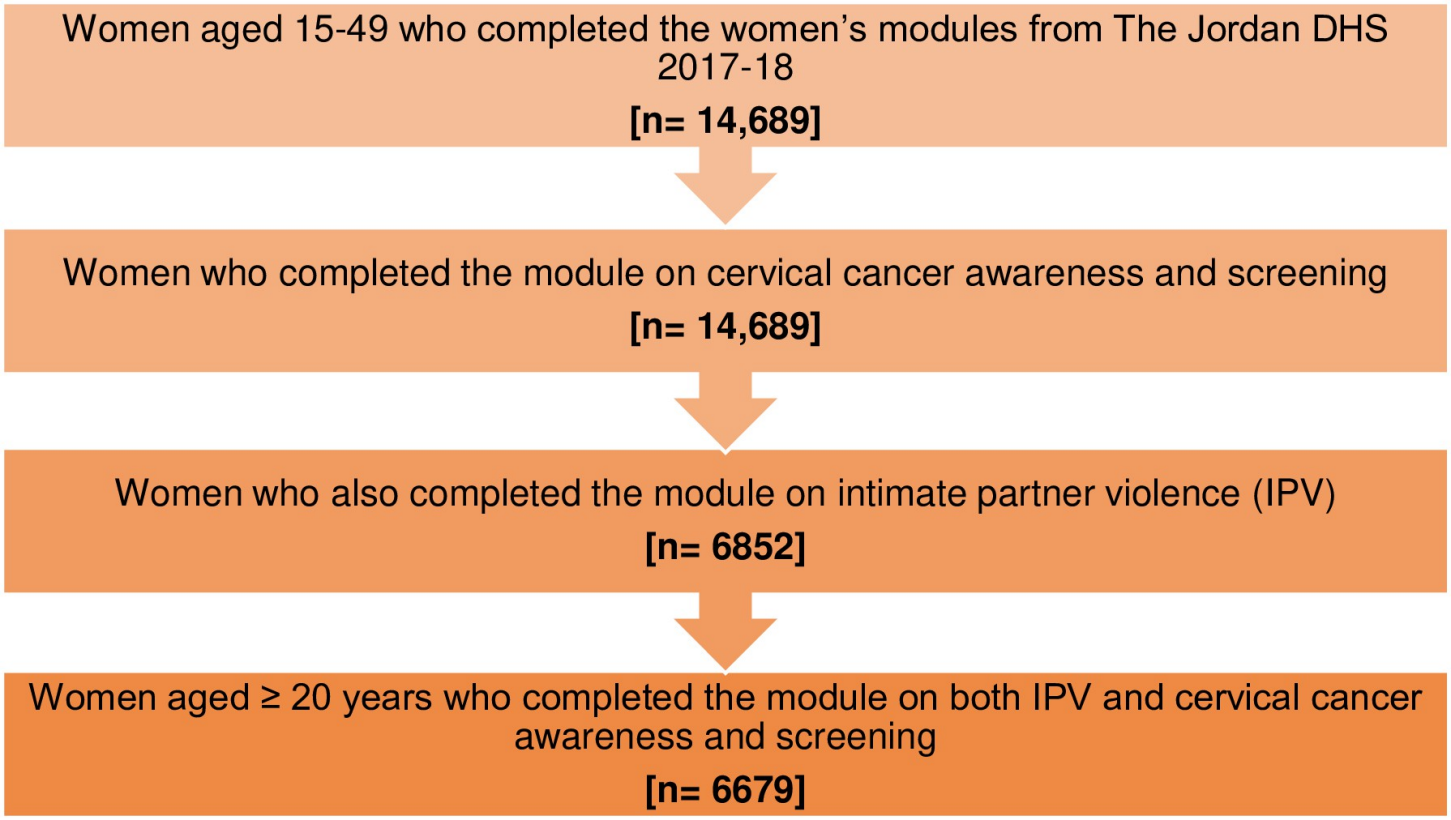
Flowchart displaying the process of determining the final sample size. Caption credit: Department of Health and Statistics. The 2017–18 Jordan Population and Family Health Survey 2017/18. Jordan Available from: https://dhsprogram.com/pubs/pdf/FR346/FR346.pdf.

14,870 eligible women were selected with 91.7% participating. All answered cervical screening awareness and uptake questions, however, only 6852 completed the IPV module as this required complete privacy. Of these, we selected only 6679 women aged 20 and above to capture Jordan’s suggested screening age (Fig 1).

### IPV measure

IPV included physical, emotional, and sexual spousal violence. Categories were recoded, combining questions detailed in the DHS survey determining exposure to violence within 12 months (12m) of the interview [14].

Physical violence was constructed into a binary variable (0 = no,1 = yes) after recoding answers to separate questions ’Did your husband ever do this to you’ with options such as ’Push you’ or ’shake you’. Answers were recoded so that ’no’ included the original values ’never’ and ’yes, but not in last 12m’ and the recoded ’yes’ included ’often’ ’sometimes’ and ’yes in last 12m’. The same process applied to emotional violence determined by answers such as ’say or do something to humiliate you in front of others?’. Sexual Violence was based on ’has your husband ever physically forced you into sexual intercourse?’ with answers recoded in the same format, ensuring the violence was captured within 12 months of interview.

Further sensitivity analysis was conducted taking into consideration ’ever’ exposure to intimate partner violence to include IPV that may have occurred any time out with the 12-month interview, increasing robustness of results.

### Ethical consideration

Data underlying the results was requested and obtained from the DHS website repository directly via a 300-word application explaining project aims. As this data was anonymised, an ethical approval was unnecessary for use, however ethical approval of the original Jordan DHS survey protocol, including biomarker collection of the DHS data, was obtained by the Department of Statistics (DOS) and was reviewed, and approved by the international coaching Federation (ICF) Institutional Review Board (IRB). The ICF IRB has strict guidelines to ensure that the original survey provided informed consent, with confidentiality ad privacy strictly adhered to. Interview or biomarker collection is carried only when the participants orally approve the narrated informed consent statement by the data collecting team.

### Outcome

Awareness of the Pap smear was determined by if a woman has ’Heard’ or ’knew’ about the Pap test. This outcome variable ’Heard of Pap smear?’ was binary (0 = no,1 = yes). Only women answering yes were asked the further question: ’Ever had a Pap smear?’ (0 = no,1 = yes) Variables included in previous research such as perceived benefits of screening were excluded from Jordan’s DHS questionnaire [15].

### Covariates

Independent variables were chosen based on the existing literature on cervical screening and included age, residence, marital status, governates, ethnicity, highest education level, wealth quintile, health insurance coverage and primary healthcare decision-maker. Governates were recoded into three regions with 1 = Northern (Irbid, Jerash, Ajloun, Mafraq), 2 = Central (Amman, Zarqa, Balqa, Madaba) and 3 = Southern (Karak, Tafielah, Ma’an, Aqaba). Ethnicity was recoded from Jordanian, Syrian, Egyptian, Iraqi, Arab and non-Arab into three choices (1 = Jordanian, 2 = Syrian, 3 = Other).

### Statistical analysis

Statistical analysis was completed using Stata version 16 with significance set at p<0.05. Categorical independent variables were analysed using the appropriate x^2^ squared tests of independence for the association. Only women illustrating the awareness of Pap smear were asked the follow-up screening question assuming that those that did not know about Pap tests would not have undergone screening. This assumption could create bias due to systemic differences between women with and without the awareness of the Pap smear test. Hence, Heckman’s two-stage probit model was applied to adjust for this section bias [30]. In Heckman’s two-stage model, awareness of Pap Smear test was the outcome variable for the selection stage. Our participants Pap smear test was the outcome variable for the outcome stage of the model. By selecting this two-stage model, we were able to address the potential bias in the sample selection that might affect our analytical model.

The Heckman’s two-stage probit selection model estimated the probability of having the Pap smear awareness by controlling for all three domains of IPV, age, place of residence, region, ethnicity, education, wealth, presence of health insurance and women’s autonomy in making decisions on their health care. The outcome model estimates the probability of undergoing a Pap smear test by adding similar variables except for the health insurance variable in the selection model. The decision to add the health insurance variable for the selection equation rather than the outcome equation is driven by the unadjusted logistic regression analysis (not shown in the paper) and the recommendation that the section model should have at least one additional explanatory variable compared to the outcome model [31]. As both the awareness and screening variables are dichotomous, we employed probit models both in the selection and outcome models.

Multicollinearity between IPV variables was assessed as well as between covariates by establishing the variance inflation factor (VIF), with no problems encountered as no value exceeded 1.5. The final stage involved weighting the data for domestic violence, with proportions updated accordingly, and original frequencies left unadjusted.

## Results

**Fig 2:**
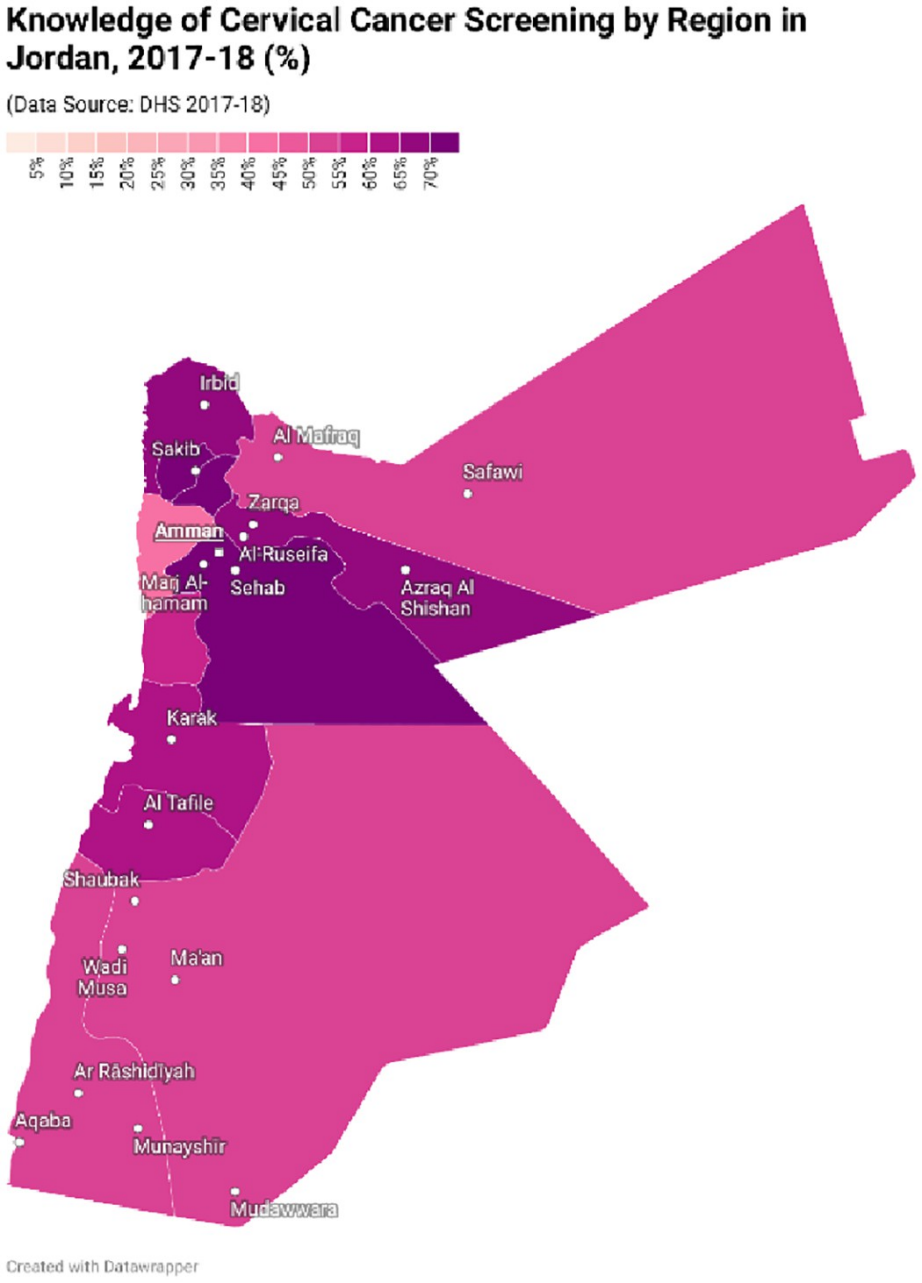
Map displaying awareness of cervical cancer screening coverage by region in Jordan, 2017–18 (%). Cervical cancer screening awareness rates are shown by region of Jordan, with higher rates depicted in a darker colour. Caption credit: Create maps (2023) www.datawrapper.de.

**Fig 3:**
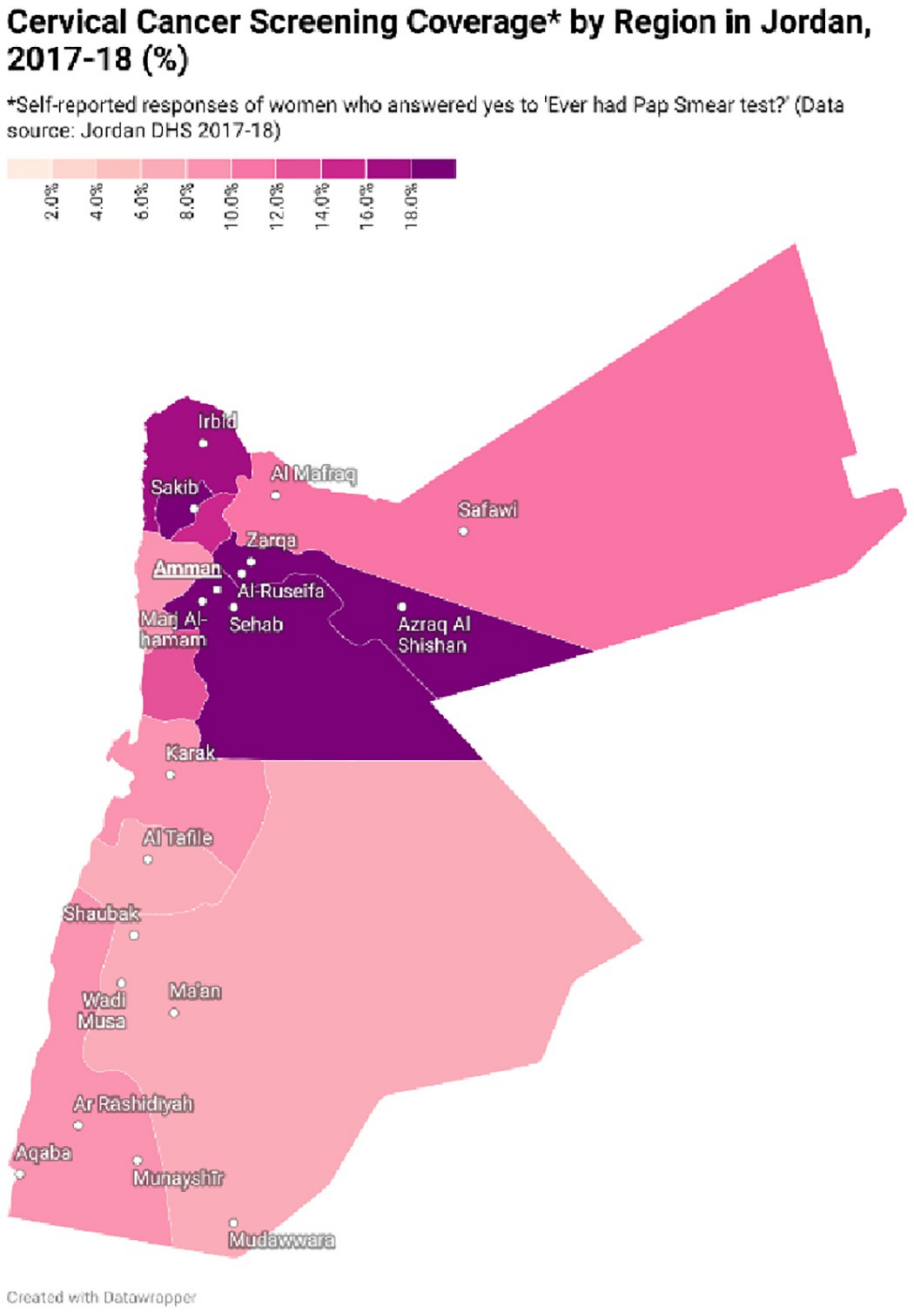
Map displaying cervical cancer screening coverage by region in Jordan, 2017–18 (%). Cervical cancer screening coverage rates are shown by region of Jordan, with higher rates depicted in a darker colour. Caption credit: Create maps (2023) www.datawrapper.de.

Population characteristics are displayed in Table 1; 65% had awareness of the Pap smear test and 15.8% had ever been screened. Screening and awareness by region in Jordan are displayed in Figs 2 and 3.

**Table 1:** Socio-economic characteristics of the women in Jordan, Demographic Health Survey 2017/18.

Characteristic	Frequency (%^a^)
**Heard of Pap smear?**	
No	2659 (35.0)
Yes	4020 (65.0)
**Had a Pap smear?**	
No	5841 (84.2)
Yes	838 (15.8)
**Age**	
20–24	715 (10.7)
25–29	1205 (18.0)
30–34	1308 (18.8)
35–39	1236 (18.6)
40–44	1112 (16.8)
45–49	1103 (17.2)
**Residence**	
Urban	5307 (90.1)
Rural	1372 (9.9)
**Marital Status**	
Married	6290 (93.1)
Widowed	177 (2.8)
Divorced/not living together/separated	212 (4.1)
**Region**	
Northern	2283 (27.7)
Central	2379 (62.7)
Southern	2017 (9.7)
**Ethnicity**	
Jordanian	5721 (86.5)
Syrian	684 (8.9)
Other Nationality	274 (4.7)
**Highest Education Level**	
No education	240 (2.1)
Primary	566 (8.4)
Secondary	3597 (53.7)
Higher	2276 (35.9)
**Wealth Quintile**	
Poorest	1812 (18.7)
Poorer	1668 (20.5)
Middle	1464 (21.1)
Richer	1109 (22.2)
Richest	626 (17.5)
**Covered by Health Insurance**	
No	1907 (41.5)
Yes	4772 (58.5)
**Person Who decides on Respondent’s Healthcare**	
Respondent alone	1310 (23.6)
Respondent and husband	4405 (68.6)
Husband /someone else alone	571 (7.7)
	**Total sample = 6679**

^a^ Numbers are unadjusted, and percentages are weighted

The population comprised 86.5% Jordanian, 8.9% Syrian and 4.7% other nationalities. 58.5% were covered by health insurance, with 18.7% in the poorest wealth index compared with 17.5% in the richest. A small proportion had no education (2.1%) with 53.7% having minimum level of secondary education. 23.6% of women made healthcare decisions independently, with 68.6% involving a husband and 7.7% of decisions were made entirely by someone else.

Table 2 shows cervical screening awareness and undertaking of cervical screening among respondents by their characteristics. All socio-economic variables were significantly associated with awareness of Pap smear tests, except residence and marital status where p>0.05 (Table 2). Furthermore, all variables other than marital status and health insurance were significantly associated with cancer screening utilisation (p<0.05).

**Table 2:** Bivariate analysis showing association between socio-economic characteristics and cervical cancer screening Jordan Demographic Health Survey 2017/18.

Characteristic	Total Sample (n = 6679)	Has awareness of cervical screening (weighted %)	P Value	Has been screened for Cervical cancer (weighted %)	P Value
**Woman’s Age**					
20–24	715	295 (44.4)	0.000	31(6.2)	0.000
25–29	1205	634 (55.7)		75 (8.1)	
30–34	1308	800 (66.1)		126 (13.4)	
35–39	1236	780 (66.9)		165 (17.4)	
40–44	1112	754 (75.7)		210 (21.8)	
45–49	1103	757 (74.0)		231 (25.1)	
**Residence**					
Urban	5307	3228 (65.4)	0.141	708 (16.2)	0.040
Rural	1372	792 (61.9)		130 (12.3)	
**Marital Status**					
Married	6290	3806 (65.4)	0.410	798 (16.1)	0.222
Widowed	177	101 (62.4)		21 (16.8)	
Divorced or not living together/separated	212	113 (58.5)		19 (9.8)	
**Region**					
Northern	2283	1468 (65.4)	0.013	349 (16.0)	0.0001
Central	2379	1404 (66.1)		338 (17.0)	
Southern	2017	1148 (57.3)		151 (7.8)	
**Ethnicity**					
Jordanian	5721	3652 (68.8)	0.000	782 (16.7)	0.025
Syrian	684	222 (38.7)		28 (9.1)	
Other Nationality	274	146 (44.3)		28 (12.4)	
**Highest Education Level**					
No education	240	77 (30.9)	0.000	15 (5.7)	0.044
Primary	566	265 (49.6)		48 (12.9)	
Secondary	3597	2192 (65.6)		502 (17.0)	
Higher	2276	1486 (69.8)		273 (15.3)	
**Wealth Quintile**					
Poorest	1812	797 (49.3)	0.000	130 (10.3)	0.000
Poorer	1668	1013 (60.8)		161 (10.4)	
Middle	1464	975 (67.0)		207 (16.4)	
Richer	1109	783 (73.8)		193 (18.6)	
Richest	626	452 (73.3)		147 (24.1)	
**Covered by health insurance?**					
No	1907	1030 (60.2)	0.000	218 (15.5)	0.719
Yes	4772	2990 (68.5)		620 (16.1)	
**Person who decides on respondent’s healthcare**					
Respondent alone	1310	837 (70.0)	0.000	174 (16.5)	0.016
Respondent & husband	4405	2678 (65.6)		565 (16.6)	
Husband/Someone else alone	571	290 (49.5)		58 (9.2)	

The largest proportion of women with awareness and history of a Pap smear test were those aged 40–44 (75.7%) and 45–49 (25.1%) respectively. Ethnicity played an important factor: 16.7% of Jordanians were tested, compared with 9.1% of Syrians. Highest uptake of Pap smear tests was attributed to a secondary level of education (17.0%), compared with 5.7% of women with no education (p<0.044). The wealthiest index had higher smear test levels (24.1%) compared to 10.3% in the poorest index (p<0.000).

In the group making independent healthcare decisions, 70.0% had test awareness and 16.5% had test experience. Healthcare decisions made solely by someone else resulted in lower levels of awareness (49.5%) and test experience (9.2%).

From the women with privacy to answer the IPV module, 180 (3.4%) were found to be victims of sexual violence, 691 of physical violence (12.6%) and 935 (16.2%) of emotional violence. A further question (not shown in the tables) asked women to rate how afraid they were of their husband. This uncovered that 8.9% of women were afraid most of the time, a concerning 51.1% were afraid sometimes, and 40% were never afraid. Furthermore, a small section of women answered questions on help-seeking behaviours from IPV (n = 1271) where only 19.6% admitted to seeking help from someone about their situation, with the remaining 80.4% of women not seeking any help at all.

Table 3 demonstrates that a statistically significant association was found between sexual violence and the awareness of cervical cancer screening (p<0.05), however, this did not influence whether women had ever been tested (p>0.05). Although emotional violence was not statistically significant in influencing awareness (Table 3), a significant association was shown with the utilisation of screening as p = 0.016 (Table 4).

**Table 3:** Bivariate analysis of the association between IPV and awareness of cervical cancer screening, Jordan Demographic Health Survey 2017/18.

Category of Intimate Partner Violence	Total Sample (n = 6679)	Does not have awareness of cervical screening (weighted %)	Has awareness of cervical screening (weighted %)	P Value
**Physical**				
No	5988	2356 (34.4)	3632 (65.6)	0.138
Yes	691	303 (38.7)	388 (61.3)	
**Emotional**				
No	5744	2262 (35.1)	3482 (64.9)	0.818
Yes	935	397 (34.5)	538 (65.5)	
**Sexual**				
No	6499	2569 (34.7)	3930 (65.3)	0.054
Yes	180	90 (43.4)	90 (56.6)	

**Table 4:** Bivariate analysis of the association between IPV and having been screened for cervical cancer, Jordan Demographic Health Survey 2017/18.

Category of Intimate Partner Violence	Total Sample (n = 6679)	Has not been screened for cervical cancer (weighted %)	Has been screened for cervical cancer (weighted %)	P Value
**Physical**				
No	5988	5238 (84.3)	750 (15.7)	0.718
Yes	691	603 (83.4)	88 (16.6)	
**Emotional**				
No	5744	5033 (85.0)	808 (15.0)	0.016
Yes	935	711 (79.8)	127 (20.2)	
**Sexual**				
No	6499	5683 (84.2)	816 (15.8)	0.878
Yes	180	158 (84.8)	22 (15.2)	

The primary focus of this study aimed to determine if IPV was a predictor of cervical cancer screening. Our Heckman Probit selection model results (Table 5) show sexual violence to be a predictor of awareness of cervical screening. Women subjected to this were significantly less likely to admit to awareness of the test compared to their non-abused counterparts (P<0.01, CI: -0.664, -0.095). However, this had no impact on access to screening services in the outcome model (p> 0.05).

**Table 5:** Heckman Probit model for predictors of awareness and uptake of Pap smear test, Jordan Demographic health survey, 2017/18.

**Has had a Pap smear test (No)** ^R^	**Outcome Model**
**Emotional Violence (No)** ^R^	
Yes	0.28 (0.0728, 0.493)**
**Physical Violence (No)** ^R^	
Yes	-0.11 (-0.346, 0.129)
**Sexual Violence (No)** ^R^	
Yes	-0.21 (-0.586, 0.159)
**Age category (20–24)** ^R^	
25–29	0.21 (-0.086, 0.497)
30–34	0.48 (0.202, 0.750)**
35–39	0.63 (0.358, 0.905)**
40–44	0.78 (0.499, 1.052)
45–49	0.83 (0.556, 1.103)
**Residence (Urban)** ^R^	
Rural	-0.03 (-0.220, 0.159)
**Region (Northern)** ^R^	
Central	-0.06 (-0.197, 0.085)
Southern	-0.41 (-0.566, -0.262)**
**Ethnicity (Jordanian)** ^R^	
Syrian	-0.12 (-0.430, 0.188)
Other Nationality	-0.02 (-0.393, 0.363)
**Highest Level of Education (None)** ^R^	
Primary	0.44 (-0.0236, 0.912)
Secondary	0.47 (0.0696, 0.867)*
Higher	0.34 (-0.066, 0.738)
**Wealth Quintile (Poorest)** ^R^	
Poorer	-0.04 (-0.227, 0.152)
Middle	0.25 (0.0430, 0.460)*
Richer	0.33 (0.118, 0.538)**
Richest	0.50 (0.261, 0.729)**
**Person Who makes respondent’s healthcare decisions (Respondent Alone)** ^R^	
Respondent and husband	0.06 (-0.856, 0.204)
Husband or someone else	-0.22 (-0.456, 0.016)
**Awareness of Pap Smear test (No)** ^R^	**Selection Model**
**Emotional Violence (No)** ^R^	
Yes	0.09 (- 0.071, 0.257)
**Physical Violence (No)** ^R^	
Yes	-0.09 (- 0.272, 0.095)
**Sexual Violence (No)** ^R^	
Yes	-0.38 (-0.664, -0.095)**
**Age category (20–24)** ^R^	
25–29	0.27 (0.095, 0.435)**
30–34	0.54 (0.363, 0.709)**
35–39	0.57 (0.392, 0.747)**
40–44	0.85 (0.669, 1.028)
45–49	0.74 (0.563, 0.914)**
**Residence (Urban)** ^R^	
Rural	-0.06 (-0.201, 0.081)
**Region (Northern)** ^R^	
Central	-0.08 (-0.205, 0.053)
Southern	-0.31 (-0.434, -0.185)**
**Ethnicity (Jordanian)** ^R^	
Syrian	-0.55 (-0.743, -0.361)**
Other Nationality	-0.40 (-0.663, -0.132)**
**Highest Level of Education (None)** ^R^	
Primary	0.76 (0.381, 1.148)
Secondary	0.90 (0.580, 1.221)
Higher	0.91 (0.580, 1.232)
**Wealth Quintile (Poorest)** ^R^	
Poorer	0.14 (-0.019, 0.304)
Middle	0.23 (0.065, 0.397)**
Richer	0.44 (0.259, 0.612)**
Richest	0.39 (0.143, 0.646)**
**Covered by health insurance? (No)** ^R^	
Yes	0.15 (0.033, 0.266)**
**Person Who makes respondent’s healthcare decisions (Respondent Alone)** ^R^	
Respondent and husband	-0.16 (-0.270, -0.043)**
Husband/ someone else	-0.38 (-0.565, -0.188)**

^R^ = Reference Category

* = P < 0.05

** = P < 0.01

Emotional violence was not a statistically significant predictor of awareness of the test but had a highly significant paradoxical association with women’s screening status (P<0.01 CI: 0.0728, 0.493). Our outcome model results conclude that Jordanian women were more likely to have undergone cervical cancer screening if subjected to emotional violence compared to those who were not emotionally abused, raising various questions.

Physical violence was not a predictor of screening in either model. It is important to note that Table 5 included violence within 12 months of the interview; however, analysis was replicated to include ’ever’ having exposure to IPV, as this could occur at any time in a woman’s life out with the interview timeframe. This analysis showed very similar results compared to our main model; hence they are not included in the table. Logistic regression analyses carried out independently (not shown in the paper) for awareness and screening outcomes showed similar results as the Heckman Probit model. Finally, a sensitivity analysis was also carried out to determine any differences in awareness and screening results between the original full sample of women (n = 14,689) and those able to answer questions on IPV (n = 6679). This confirmed no statistically significant difference between the two groups, therefore increasing the robustness of results.

Results of the selection model showed that women were less likely have awareness of screening if their healthcare decisions were made by someone else compared to independently (P<0.01, CI: -0.565, -0.188).

Residence was insignificant, implying those living rurally were not disadvantaged compared to urban settings. Health insurance was shown to be a predictor of awareness. Syrian women were less likely to have heard of cervical cancer screening compared to Jordanian women as was also the case for ’other nationalities’ (P<0.01 CI: -0.743, -0.361 and -0.40 (P<0.01 CI: -0.663, -0.132). However, ethnicity was not a predictor of screening as shown in the outcome model. Women with secondary education were more likely to be screened than those with no educational background (P<0.05, CI: 0.0696, 0.867). Women from middle, richer and richest wealth quintile had more awareness compared to the poorest women (p<0.01, CI: 0.065, 0.397 and p<0.01, CI: 0.259, 0.612 and p<0.01, CI: 0.143, 0.646). Similarly, screening rates were higher in the middle, richer and richest women compared to poorest (p<0.05, CI: 0.0430, 0.460 and p<0.01, CI: 0.118, 0.538 and p<0.01 CI: 0.261, 0.729). Also, women from Southern Jordan had lower level of awareness and screening rates in comparison to Northern women (p<0.01, CI: -0.434, -0.185 and p<0.01, CI: -0.566, -0.262 respectively).

## Discussion

This paper is the first of its kind to use national-level data in this context to identify associations between cervical cancer screening and IPV in Jordan, a country where lower level of awareness (65%) and screening rates (15.8%) were detected. The results concluded that women subjected to sexual violence were less likely to admit to having awareness of a Pap smear test; however, this did not impact screening rates. Furthermore, victims of emotional violence, paradoxically, were more likely to be screened for cervical cancer than non-victims. No association between physical violence and cervical cancer screening was found.

Low screening rates are a common finding in Arab countries, widely observed due to limited resources directed towards the development of comprehensive cervical cancer screening programmes. No Arab country has a call-and-recall invitation system similar to Europe [32] which is proven to reduce cervical cancer mortality [33, 34]. For example, a study from Iraq found that only 32.4% of women had adequate awareness and 12.6% of women had been screened [35]. Similarly, a Saudi Arabian study identified that only 33.4% of women had been screened for cervical cancer [36] and a study from Kuwait found only 52% of women to have adequate awareness with 23.8% of women screened [37]. These findings reflect the common misconception that screening is culturally unacceptable for Muslim women as Islam prohibits premarital sexual intercourse, therefore in this conservative culture, HPV associated with promiscuity is not considered a risk factor [7, 38–41].

The complex relationship between the association of sexual violence with lower level of awareness of cervical screening may be explained by the admission of screening knowledge acting as a precursor to suggestions of health services engagement. Despite sexual violence influencing awareness of the Pap smear test, our study did not show any association between exposure to sexual violence and actual cervical cancer screening rates. The rationality behind this relationship is hard to determine due to limited questions in the DHS survey. This was an unexpected finding, despite the evidence that sexual violence can act as a barrier to healthcare access and, subsequently, inadequate screening [42–44].

However, similar to our findings, one American study described that victims of sexual violence under 40 years old did not report statistically different cervical cancer screening rates compared to the general population. Victim status may not play a part as screening can occur opportunistically during family planning services in this reproductive age group [45]. Furthermore, another study found no difference in sexual and physical violence victims in receipt of cervical cancer screening compared to the general population [41]. A recent meta-analysis of 36 studies concluded that all three forms of IPV again were not related to cancer screening practices but, worryingly, were significantly associated with the incidence of abnormal Pap smear test results and, therefore, greater odds of cervical cancer [46].

Also, studies showed that for victims of sexual violence, the Pap smear test is an invasive procedure identified as a re-traumatising experience uncovering evidence of assault a survivor is trying to hide, with an expectation of pain and associated fear or embarrassment [45, 47, 48]. Therefore, women may avoid the test despite having a higher risk for cervical cancer due to IPV exposure. Besides, women who undergo sexual violence in Jordan may experience it as a teenager due to their young marital age, further limiting their agency to accessing information on sexual health, including cervical cancer screening [49]. Child brides are less likely to access healthcare due to decreased agency and bargaining power [50]. The legal age of marriage in Jordan is 18; however, children aged 15–17 can be married under exceptional circumstances. UNICEF express concerns that child marriage is increasing, especially within Syrian refugee communities, with a reported 36% of all Syrian marriages in Jordan involving children [51, 52]. In our study, the odds of having awareness of cervical cancer screening and undergoing screening increased with age from 25 onwards. As well as having lower level of awareness of screening, a decade of trends shows that younger brides have an increased risk of IPV, which consequently impacts on their autonomy [51, 52].

Physical violence was not shown to be statistically significant in either model. This was inconsistent with a Brazilian study’s findings demonstrating an association between physical IPV and inadequate cervical cancer screening [48]. Similarly, a second Brazilian study showed an association between physical and sexual IPV and lower rates of this screening [53].

Emotional violence frequently occurs in Jordan’s patriarchal society where male privilege leads to intimidation and dominance [54, 55]. It is often reported that emotional violence can be insidious, resulting in chronic suffering, leaving a woman vulnerable, anxious and with low self-esteem [51]. In our study, victims of emotional violence were paradoxically more likely to be screened for cervical cancer than those participants who were not exposed to emotional abuse. We argue that the results, rather than suggesting that emotional violence is beneficial for screening, indicate a lack of reliability of questions as well as the response of the participants. These controversial findings were also reported by a few other studies. For instance, a study focusing on women experiencing emotional abuse were shown to have more frequent consultations in a North of England hospital. In particular, those who underwent emotional abuse had more worries about smear abnormality and cancer than their non-abused counterparts [56]. While the authors did not want to speculate the reasons for this association, they suggested that the significant association of emotional abuse with higher levels of anxiety could result in physical symptoms. They cited a study [57] that suggested that majority of women who experienced emotional abuse had physical symptoms including headaches, chronic pain and vaginal bleeding.

A study in Vienna found that women who experience all three modes of violence reported higher odds of gynaecological symptoms and therefore have more visits to healthcare providers [58]. The authors argued that worry about health was mediating the association between violence and gynaecological symptoms. It is likely that in our sample, women experiencing emotional violence were more likely to act on their worries to visit health care for screening. One study found that victims of sexual and physical abuse aged 40 and above were 87% less likely to have had Pap smears compared to those who had been emotionally abused [45]. Contrary to this study, we did not compare cervical cancer screening rates of victims of emotional abuse directly with those experiencing sexual and/or physical abuse. However, both these studies suggest that women experiencing emotional violence have higher levels of screening compared to either those that did not experience emotional abuse or compared to those experiencing sexual and/or physical abuse. However, the authors were unable to explain why victims of emotional abuse had the highest rates of cervical cancer screening compared to those experienced sexual and/or physical abuse. While we also find this association complex, we explored the reasons by carrying out further analysis on justification of various domains of abuse. Our analysis (not reported in the tables) found that 87% of emotional violence survivors said that beating is not justified if the wife argues with her husband. This was higher than those who experienced physical violence, where 84% said it was not justified, and 82% for sexual violence survivors. This may strengthen the hypothesis that victims of emotional abuse might have stronger autonomy in this context, and therefore are able to speak up and present more frequently to healthcare providers.

The other plausible explanation could be related to the measurement of various modes of IPV and the importance given to them. Often several researchers combined all domains of IPV rather than looking at various domains individually [21, 59, 60]. This has limited the ability to compare our study with the findings measures in a singular format. Often psychological violence has been ignored in LMIC research. This could mean that there might still be challenges to the measurement of this component in countries where research on psychological or emotional abuse is in the nascent stages [27]. Alternatively, women might not hide psychological abuse and might hide sexual and physical violence due to the stigma associated with them. Besides, it is hard to understand the association and the pathways without measuring the severity of emotional abuse in relation to the frequency.

There is emerging research on ’reproductive coercion’ within violent partner relationships where men control a woman’s reproductive health access and decisions [61, 62]. Women may feel coerced into making healthcare decisions about important family planning which may lead to an unwanted termination of pregnancy or family size [63]. This is validated in our findings which demonstrated that if a women’s healthcare decisions were made entirely by the husband/partner/someone else, she was less likely to have awareness of cervical cancer screening.

Ethnicity played a role in the awareness of the Pap smear test; Syrian women were less likely to have awareness of screening compared to their Jordanian counterparts. It is important to consider that Jordan currently hosts 670,000 Syrian refugees—80% are under the poverty line [64]. These findings may reflect free universal refugee health coverage ending in 2014 due to a decline in physicians per person resulting from the influx of Syrian refugees [65]. Our findings found women with health insurance coverage and increasing wealth quintile were associated with higher odds of awareness of cervical cancer screening. Therefore, Syrian refugees disproportionately face barriers to accessing healthcare [66]. It was unclear how many of our sample had refugee status; however, 3.9% used refugee health insurance.

It is universally recognised that women living with intimate partner violence are a vulnerable subgroup that must not be overlooked. It should be acknowledged that the situation may be far worse than this study reports, as women without autonomy were unlikely to answer questions that may endanger them. Therefore, a large subgroup of women are not accounted for. Societal restrictions imposed by the Covid19 pandemic may have exacerbated this situation further.

It has been suggested in previous research that Jordanian women may fail to disclose IPV in the absence of current injuries and that Jordanian medical facility staff require training in effective IPV screening methods [67]. Jordan’s health system complexity, with health insurance providers ranging from the United Nations Refugee Welfare Association to private insurance [14], means creating a standard training framework is challenging, yet a priority.

### Limitations

The sensitive nature of the questions introduces uncertainty in the accuracy of IPV representation in the sample. Women may feel worried about answering honestly, opening wider questions about capturing delicate information in qualitative surveys. These under-reporting challenges were outside of the study’s control. The DHS survey questions were not designed for cervical cancer screening and subsequently did not provide detailed information on full Pap smear history, instead only if they have ’ever’ been screened. However, it allowed a general nation-level estimate of the current situation.

### Implications for future research

As previously mentioned, the WHO now recommends the use of HPV testing over the conventional Pap smear test that is standard practice in Jordan and many other non-European countries [68]. Most recently in 2021, this has been adapted to involve self-sampling as an approach to target women who may not engage with clinician-based interventions and is particularly beneficial in low-resource settings where there are high populations of women who have not been screened [69, 70].

Women who have faced barriers to screening, such as fear, pain, embarrassment, or avoidance due to previous sexual or physical violence, may benefit from this new method of self-sampling as it puts them in control of the process. For women who are subject to IPV and experience the constraints of controlled access to healthcare by their partner, this option may theoretically act as a more discrete method of testing; however, it would still require access to health services to collect and return the sample.

Our research has highlighted the necessity to improve public health promotion of cervical cancer screening amongst the population of Jordan, alongside targeting the women who are vulnerable to underscreening. A suggested approach may begin with appropriate education of health care workers in Jordan, as studies have identified discrepancies in awareness and understanding of cervical cancer screening tests available. One study found that only half of healthcare professionals were considered aware of cervical cancer screening [39]. Another Jordanian study found 20% of ObGyn clinicians did not think HPV was involved in cervical cancer aetiology, and more than half voiced opinions that the Pap Smear was not the most cost-effective public health tool for cancer screening [40].

Therefore, we implore the Jordanian government to address and identify these gaps in awareness and understanding within both the Jordanian population and healthcare workers surrounding the most appropriate and cost-effective method of screening.

## Conclusion

Our study examined the association between IPV and cervical cancer, the first to our knowledge to use nationally representative data for Jordan for this purpose. Our research concludes that while sexual violence is associated with cervical cancer screening awareness, emotional violence is associated with increased rates of screening in Jordan, an important and complex finding warranting further research. Based on this, we recommend developing qualitative methods to capture the full population of women at risk of IPV and tailored cervical cancer questions to understand the situation’s complexity. We also suggest that Jordanian healthcare professionals improve the integration of reproductive health services with IPV screening, ensuring vulnerable women are identified and safeguarded.
